# An analysis of perceptions and thoughts of rapid weight loss in Olympic weightlifters

**DOI:** 10.1177/02601060241305478

**Published:** 2024-12-12

**Authors:** Thomas I Gee, Paul Campbell, Melissa J Bargh, Daniel Martin

**Affiliations:** School of Psychology, Sports Science, and Wellbeing, 4547 University of Lincoln, Lincoln, UK

**Keywords:** Weight-cutting, Olympic weightlifting, mood, rapid weight loss, athletes

## Abstract

**Background:**

Weight-class athletes commonly engage in rapid weight loss (RWL) practices.

**Aim:**

Investigate attributed RWL perceptions and thoughts of UK-based Olympic weightlifters.

**Methods:**

Participants (n: 39, male: 22, female: 17) were selected from International Weightlifting Federation populations, 85% had previously acutely reduced pre-competition body mass. The ‘Pre-competition weight management practices questionnaire’ featured open-ended questions concerning feelings, mood and thoughts during RWL. Qualitative responses were subsequently analysed using content analysis principles.

**Results:**

Negative thoughts and perceptions were commonly attributed concerning RWL, of which codes: irritation, hunger, fatigue, anxiety and low mood accounted for 72% of the 72 data extracts. Females more frequently attributed codes of anxiety, hunger and low mood.

**Conclusion:**

The prevalence of negative thoughts and perceptions of RWL accord to previous reports within strength-sport athletes. Olympic weightlifting athletes and coaches should contemplate if negative feelings/mood perceived during RWL are a tolerable factor within pre-event preparation to compete in a desired weight class.

## Introduction

In respect to competitive weight-class sports, rapid weight loss (RWL) has been described as a body mass reduction of 2–10% generally within 2–3 days of weigh-in (Artioli et al., 2016). Also termed ‘weight cutting’, RWL to compete within a chosen weight class, has been reported within strength sports (Campbell et al., 2023; Gee et al., 2023; Nolan et al., 2022). Past research within powerlifters have reported a high prevalence of RWL (86–97%), and a moderate body mass loss before competition weigh-in (3–4%) (Campbell et al., 2023; Kwan and Helms, 2022; Nolan et al., 2022). Accordingly, we recently reported a pre-competition habitual body mass loss of 3.8% within Olympic weightlifters (Gee et al., 2023). Review articles investigating RWL have reported that acute body mass losses above 3% have resulted in impairments within high-intensity exercise performance and detrimental effects on aspects of physical health such as acute cardiovascular strain, hormonal imbalances and suppressed immune function (Barley et al., 2019; Brechney et al., 2022). Within strength-sports, principal governing bodies, for instance the International Weightlifting Federation (IWF) and International Powerlifting Federation (IPF) implement a <2 h weigh-in before competition. This limits the scope for athletes to engage in ensuing hydration and rapid weight gain and therefore recovery from such RWL, potentially exposing the athlete to deleterious effects on performance and health during competition (Campbell et al., 2023; Gee et al., 2023).

Previous authors have emphasised the need for subsequent qualitative research within strength-sport athletes to explore pressures and motivations experienced regarding RWL (Campbell et al., 2023; Wood et al., 2022). We recently published the initial investigation to feature open-ended questioning regarding competitive powerlifters’ mood, feelings and thoughts of RWL (Campbell et al., 2023). Following inductive analysis there was the emergence of three categories in respect to participants responses: emotional responses, cognitions and physiological perceptions. We reported primarily unfavourable perceptions of RWL with codes of as low mood, irritation, anxiety, sensations and fatigue accounting for ∼70% of responses. Although, a lower sample of responses reported ‘focus’ (17%) and ‘positive mood’ (9%) during weight cutting, which indicates a goal-oriented approach in regards to weigh-in body mass loss (Pettersson et al., 2012). Another previous investigation examined the prevalence of five perceived psychological states (depression, anxiety, isolation, anger and fatigue) within powerlifters who engaged within RWL, however using a categorical questioning approach, as opposed to inductive qualitative ‘open-ended’ questioning (Kwan and Helms, 2022). Since a proportion of powerlifting athletes reported positive states via our previous inductive analysis, this indicates that a focus on purely categorical negative states is a somewhat limited approach (Campbell et al., 2023; Kwan and Helms, 2022). Indeed elite combat sport athletes from judo, wrestling and taekwondo perceived weight cutting to be a key component of the culture of the sport (Pettersson et al., 2013). Participants discussed feelings of being ‘focused’, ‘maximally prepared’, ‘having an edge’ and a ‘mental advantage’ (Pettersson et al., 2013). Therefore to understand and conceptualize both negative and positive perceptions of RWL, an inductive qualitative approach is favoured. We recently published the first study to report prevalence and magnitude of RWL within an cohort of Olympic weightlifters, however there is currently a lack of research regarding qualitative perceptions associated during RWL within Olympic weightliters (Gee et al., 2023; Nelson and Jette, 2023). This is in spite of previous unfavourable impacts on ‘general well-being’, ‘emotional stress’ and ‘fatigue’ measured through REST-Q and POMS being reported within Olympic weightlifters following a 6-day period of purposeful bodyweight loss prior to a simulated competition (Durguerian et al., 2016). Nelson and Jette (2023) proposed that Olympic weightlifting could be described as having a culture of weight loss, in regards to the weight management practices of many lifters including pre-competition RWL. Across 16 competitive weightlifters the authors interviewed, the dominant narrative framed by the authors was that ‘Muscle moves mass’. This narrative views that only muscle mass as having a link to maximal strength and potentially causes weightlifters to habitually avoid weight gain for fear of gaining body fat. The authors postulated that the ‘Muscle moves mass’ narrative provides a framework for the culture of weight loss and the acceptance and practice of weight cutting within weightlifters.

This study aimed to investigate attributed perceptions and thoughts of UK-based Olympic weightlifters who practiced pre-competition RWL.

## Methods

### Participants

Participants (n: 39, male: 22, female: 17) lifted within ‘British Weightlifting’ sanctioned competitions (UK's IWF affiliate association, mandatory 2-h pre-competition weigh-in). Convenience sampling was used to recruit participants following initial correspondence with coaches based at numerous Olympic weightlifting clubs within the UK. Subsequent to this point of access, the authors attended training sites and competitions sanctioned by British Weightlifting during which participants volunteered to take part in the study. Participants were eligible for inclusion within the study if they had competed within a British Weightlifting or IWF sanctioned competition within the 12 months prior to survey completion. The age range of participants was 19–43 years. The participants who were found to engage in RWL were then grouped via gender (male, female).

### Questionnaire

The featured questionnaire titled ‘Pre-competition weight management practices questionnaire’ featured open-ended questions regarding mood, feelings and thoughts during the process of RWL and post weigh-in. Regarding qualitative questioning aspects of the questionnaire, participants were asked the following questions:

Please describe your main thoughts, feelings and general mood during the weight cutting process?

Do your main thoughts, feelings and general mood remain post weigh-in? YES / NO If the answer was YES, please comment on how and when they affect you

Before completing the survey, participants were required to read and sign an institutionally approved informed consent document. By which, participants were informed that they were able to ask the researchers questions and have these answered satisfactory, that their personal details would be kept confidential and that they were free to withdraw from the study at any time. Approval was granted for the study by the local ethics committee in accordance with Helsinki Declarations.

### Data analysis

Content analysis and thematic analysis principles were used to analyse qualitative responses (Clarke and Braun, 2017; Miles and Huberman, 1994). Each qualitative response given was read and re-read by the authors to enhance familiarity with the data (Maykut and Morehouse, 1994). Subsequently, following an inductive approach, codes were generated by identification of relevant data extracts. Categories were then established from the generated codes based on the frequency of occurrence of keywords which captured the wider meaning of the data and to illustrate consistency in the responses. A ‘critical friend’ approach was then subsequently adopted, which involved a critical dialogue between authors, whereby each authors data interpretations were discussed and critical feedback was offered by co-authors (Smith and McGannon, 2018). This resulted in changes to some codes/categories, by the process of critical challenge by authors to reduce the potential for data misinterpretation and error.

## Results

Mean age of the sample (*n* = 39) was 28 ± 6 years, competitive experience was 4 ± 3 years, ‘Sinclair coefficient total’ was 239 ± 60, which represented competitive standard . The mean number of competitions participants had competed in a mean of 3 ± 2 competitions during the 12 months before survey completion. Of responses, 33 of 39 (85%) participants had previously acutely reduced body mass for a competition, a higher proportion evident within the recruited females (16/17; 94%) compared to males (17/22; 77%).

A total of 32 out of 33 weightlifters (male; 16, female; 16) who had previously engaged in RWL responded to the featured open-ended questions. Content analysis of these responses revealed 72 raw data extracts which resulted in 16 codes being generated (see Table 1). Codes were then aligned to categories: reflections, emotional response, physiological perceptions and cognitions. Emotional responses were the most frequently indicated category, with low mood (53%) being most prevalent (Table 2). Additionally, codes for hunger (31%), fatigue (28%), irritation (25%) and anxiety (25%) were observed with notably high frequency. Females more frequently attributed low mood (m: 7, f: 10), hunger (m: 3, f: 7) and anxiety (m: 3, f: 5) compared to males. However, only a small proportion of the total sample (7/32; 22%) indicated that attributed feelings, general mood and thoughts remain following weigh-in.

**Table 1.** Frequency of codes as identified via content analysis and assigned categories.

Emotional response	
Low mood	n: 17 (m: 7, f: 10)
Irritation	n: 8 (m: 4, f: 4)
No change to mood	n: 5 (m: 3, f: 2)
Positive mood	n: 4 (m: 1, f: 3)
**Cognitions**	
Anxiety	n: 8 (m: 3, f: 5)
High degree of challenge	n: 4 (m: 2, f: 2)
Focus	n: 3 (m: 2, f: 1)
Low degree of challenge	n: 2 (m: 1, f: 1)
Distraction	n: 2 (m: 0, f: 2)
Dissociation	n: 1 (m: 1, f: 0)
Lack of confidence	n: 1 (m: 1, f: 0)
High confidence	n: 1 (m: 0, f: 1)
**Physiological perceptions**	
Hunger	n: 10 (m: 3, f: 7)
Fatigue	n: 9 (m: 4, f: 5)
High energy	n: 1 (m: 1, f: 0)
**Reflections**	
Poor preparation	n: 3 (m: 1, f: 2)

Note: n: overall (male: n, female: n).

**Table 2.** Olympic weightlifters attributed feelings, mood and thoughts of RWL, codes with a frequency of 4 >  are listed.

Example quotes	Code	Category
‘Sad – very sad! Very angry too!.’ (Female)	Low mood	Emotional response
‘Enjoyment of food is taken away. I restrict my bread intake and as a lover of bread this is just a bit upsetting.’ (Female)		
‘Generally moody or “hangry”.’ (Male)		
‘Frustration a day before competing.’ (Male)	Irritation	
‘Generally quite …short tempered.’ (Female)		
‘Going to the toilet a lot was uncomfortable.’ (Male)		
‘Doesn’t affect me mentally.’ (Male)	No change to mood	
‘I generally tend to be ok.’ (Female)		
‘Hasn’t up until this point affected me much.’ (Female)		
‘My mood is generally pretty good.’ (Female)	Positive mood	
‘Happy and positive.’ (Male)		
‘Generally happier when well hydrated and not excessive gaps between eatings.’ (Female)		
‘Closer the comp gets the more my anxiety increases.’ (Female)	Anxiety	Cognitions
‘Occasionally I get stressed about decreased performance.’ (Male)		
‘During last 36 hours (no food) and final 8 hours (no liquids) I’m…anxious.’ (Female)		
‘The last 24 h can be difficult if having to sauna.’ (Female)	High challenge	
‘Was hard to drink a lot of water.’ (Male)		
‘Days out difficult to manage. It's a tough process.’ (Female)		
‘I do find myself thinking about food more often.’ (Female)	Hunger	Physiological perceptions
‘I was hungry before almost every meal.’ (Male)		
‘2 weeks from competition I feel …ravenous.’ (Female)		
‘Body feels tired.’ (Male)	Fatigue	
‘Not very nice, tired.’ (Male)		
‘Feelings of fatigue.’ (Female)		

## Discussion

Prevalence of pre-competition weigh-in RWL is high within the sample of Olympic weightlifters (85%). This is in accordance with levels of RWL prevalence within prior samples of powerlifters who adhered to the same 2-hour pre-competition weigh-in regulations (86–97%) (Campbell et al., 2023; Kwan and Helms, 2022; Nolan et al., 2022). Further details regarding the comparative magnitude of RWL within the current sample are described within our previous article (Gee et al., 2023). The current study featured open-ended questions in regards to Olympic weightlifters’ feelings, general mood and thoughts of RWL. The primary emerging categories are from responses of emotional responses, cognitions and physiological perceptions and are in accordance with our findings within powerlifters (Campbell et al., 2023). The inductive analysis revealed a predominance of negative perceptions and thoughts of RWL, with codes of irritation, hunger, fatigue, anxiety and low mood representing a high proportion of data extracts (72%). These perceptions are indicative of the extent of reported pre-competition habitual RWL within the sample, which we previously reported to be 3.8% (Gee et al., 2023). In addition to mood state disruptions, acute body mass losses above 3% have been associated with resultant decreases in high-intensity exercise performance, and acute cardiovascular strain, hormonal imbalances and suppressed immune function (Barley et al., 2019; Brechney et al., 2022). Similarly, within our previous investigation of RWL practices within powerlifters, analysis also communicated a predominance of unfavourable perceptions of RWL with codes of; anxiety, irritation, low mood, sensations and fatigue prevalent within ∼70% of responses (Campbell et al., 2023). In addition, prominence of these negative mood states during weight cutting have been observed within combat athletes, such as MMA fighters (Brandt et al., 2018), amateur boxers (Hall and Lane, 2001) and judoists via validated psychological questionnaires (POMS and BRUMS) (Yoshioka et al. 2006). Furthermore, negatives effects on states of emotional stress, general well-being and fatigue assessed via REST-Q and POMS were reported within a cohort of Olympic weightlifters who reduced body mass over six days before a simulated competition (Durguerian et al. 2016).

Interestingly, low mood (m: 7, f: 10), hunger (m: 3, f: 7) and anxiety (m: 3, f: 5) were more frequently attributed within females compared to males. The higher frequency of response for these factors within the female weightlifters may be related to the extent of pre-competition body mass loss that the female cohort had experienced, since females attributed a significantly greater relative highest competition body mass loss compared with males (7.4 vs 4.9%), as reported in our prior investigation (Gee et al., 2023). Low mood and anxiety codes followed a similar pattern to our previous inductive analysis within powerlifters (low mood m = 4, f = 6; anxiety m = 3, f = 6) (Campbell et al., 2023). Umeda et al. (1999) reported significant increases in ‘confusion’ and ‘state anxiety’ for females during weight cutting in Judo athletes, however levels were unchanged in males, although both sexes experienced similar decreases in ‘vigor’. Within the present investigation a lower proportion of responses attributed ‘positive mood’ (n = 4) and ‘focus’ (n = 3) during RWL. Previously Pettersson et al. (2013) outlined positive perceptions of pre-competition weight regulation within elite combat sport athletes such as being ‘focused’, ‘maximally prepared’, ‘having an edge’ and a ‘mental advantage’. The positive orientation of these responses are indicative of a goal-oriented focus to pre weigh-in body mass loss (Pettersson et al., 2012). These perceptions were expressed more commonly amongst the cohort combat athletes compared to the weightlifters within the current study (Pettersson et al. 2013). However, weight cutting is likely currently viewed as a more prominent aspect of combat sport culture compared to strength sports, even though certain RWL behaviours/actions within fighters have been labelled as alarming from a health perspective and raised concern across authors (Crighton et al., 2016; Pettersson et al. 2013; Zhong et al., 2024). Although recently, Nelson and Jette (2023) proposed that Olympic weightlifting could be described as having a culture of weight loss, more research across competitive weightlifting cohorts is required to confirm such a notion.

In conclusion, in the current study the reported unfavourable mood effects attributed by Olympic weightlifters for pre weigh-in RWL, and those within our previous work within powerlifters (Campbell et al., 2023) are concerning, given such that the ability to produce appropriate pre-competition emotional feeling has been recognised as a crucial factor contributing to athletic performance (Durguerian et al., 2016). Improved access to qualified guidance such as via nutritionists and sports psychologists would likely be of benefit to competitive weightlifters concerning weight cutting and associated mood effects. In addition, investigations exploring more in-depth accounts of Olympic weightlifters experience with RWL via interviews or working groups could provide more insight regarding weight making practices. Olympic weightlifting athletes and coaches should contemplate if negative feelings/mood perceived during RWL are a tolerable factor within pre-event preparation to compete in a desired weight class.

## References

[bibr1-02601060241305478] Artioli GG Saunders B Iglesais RT , et al. (2016) It is time to ban rapid weight loss from combat sports. Sports Medicine 46(11): 1579–1584. 27102173 10.1007/s40279-016-0541-x

[bibr2-02601060241305478] Barley OR Chapman DW Abbiss CR (2019) The current state of weight-cutting in combat sports. Sports (Basel) 7(5): 123. 31117325 10.3390/sports7050123 PMC6572325

[bibr3-02601060241305478] Brandt R Bevilacqua GG Coimbra DR , et al. (2018) Body weight and mood state modifications in mixed martial arts: An exploratory pilot. Journal of Strength and Conditioning Research 32(9): 2548–2554. 29927894 10.1519/JSC.0000000000002639

[bibr4-02601060241305478] Brechney GC Cannon J Goodman SP (2022) Effects of weight cutting on exercise performance in combat athletes: A meta-analysis. International Journal of Sports Physiology and Performance 17(7): 995–1010. 35523423 10.1123/ijspp.2021-0104

[bibr5-02601060241305478] Campbell P Martin D Bargh MJ , et al. (2023) A comparison of rapid weight loss practices within international, national and regional powerlifters. Nutrition and Health. Online ahead of print. 10.1177/02601060231201892 PMC12174615 37697737

[bibr6-02601060241305478] Clarke V Braun V (2017) Thematic analysis. The Journal of Positive Psychology 12(3): 297–298.

[bibr7-02601060241305478] Crighton B Close GL Morton JP (2016) Alarming weight cutting behaviours in mixed martial arts: A cause for concern and a call for action. British Journal of Sports Medicine 50(8): 446–447. 26459278 10.1136/bjsports-2015-094732

[bibr8-02601060241305478] Durguerian A Bougard C Drogou C , et al. (2016) Weight loss, performance and psychological related states in high-level weightlifters. International Journal of Sports Medicine 37(3): 230–238. 26701827 10.1055/s-0035-1555852

[bibr9-02601060241305478] Gee TI Campbell P Bargh MJ , et al. (2023) Rapid weight loss practices within Olympic weightlifters. Journal of Strength and Conditioning Research 37(10): 2046–2051. 37729517 10.1519/JSC.0000000000004507

[bibr10-02601060241305478] Hall CJ Lane AM (2001) Effects of rapid weight loss on mood and performance among amateur boxers. British Journal of Sports Medicine 35(6): 390–395. 11726472 10.1136/bjsm.35.6.390 PMC1724425

[bibr11-02601060241305478] Kwan K Helms E (2022) Prevalence, magnitude, and methods of weight cutting used by world class powerlifters. Journal of Strength and Conditioning Research 36(4): 998–1002. 35319002 10.1519/JSC.0000000000004199

[bibr12-02601060241305478] Maykut P Morehouse R (1994) Beginning Qualitative Research – A Philosophic and Practical Guide. London: Falmer Press.

[bibr13-02601060241305478] Miles MB Huberman AM (1994) Qualitative Data Analysis: An Expanded Sourcebook. 2nd ed Thousand Oaks, CA: Sage Publications.

[bibr14-02601060241305478] Nelson M Jette S (2023) Muscle moves mass: Deconstructing the culture of weight loss in American Olympic weightlifting. International Review for the Sociology of Sport 58(5): 765–782.

[bibr15-02601060241305478] Nolan D Lynch EL Egan B (2022) Self-reported prevalence, magnitude and methods of rapid weight loss in male and female competitive powerlifters. Journal of Strength and Conditioning Research 36(2): 405–410. 31904717 10.1519/JSC.0000000000003488

[bibr16-02601060241305478] Pettersson S Ekström MP Berg CM (2013) Practices of weight regulation among elite athletes in combat sports: A matter of mental advantage? Journal of Athletic Training 48(1): 99–108. 23672331 10.4085/1062-6050-48.1.04 PMC3554040

[bibr17-02601060241305478] Pettersson S Pipping Ekström M Berg CM (2012) The food and weight combat. A problematic fight for the elite combat sports athlete. Appetite 59(2): 234–242. 22609334 10.1016/j.appet.2012.05.007

[bibr18-02601060241305478] Smith B McGannon KR (2018) Developing rigor in qualitative research: Problems and opportunities within sport and exercise psychology. International Review of Sport and Exercise Psychology 11(1): 101–121.

[bibr19-02601060241305478] Umeda T Nakaji S Sugawara K , et al. (1999) Gender differences in physical and psychological stress responses among college judoists undergoing weight reduction. Environmental Health and Preventive Medicine 4(3): 146–150. 21432188 10.1007/BF02932271 PMC2723525

[bibr20-02601060241305478] Wood TJ Wilson LJ Curtis C (2022) Quantifying frequency of use of methods of body mass loss in competing UK powerlifters. Performance Enhancement & Health 10(2): 100221.

[bibr21-02601060241305478] Yoshioka Y Umeda T Nakaji S , et al. (2006) Gender differences in the psychological response to weight reduction in judoists. International Journal of Sport Nutrition and Exercise Metabolism 16(2): 187–198. 16779925 10.1123/ijsnem.16.2.187

[bibr22-02601060241305478] Zhong Y Song Y Artioli GG , et al. (2024) The practice of weight loss in combat sports athletes: A systematic review. Nutrients 16(7): 1050. 38613083 10.3390/nu16071050 PMC11013344

